# Estimating scenarios for survival time in patients with advanced melanoma receiving immunotherapy and targeted therapy

**DOI:** 10.1093/oncolo/oyae089

**Published:** 2024-05-20

**Authors:** Megan Smith-Uffen, John Park, Andrew Parsonson, Belinda E Kiely, Anuradha Vasista

**Affiliations:** Department of Medicine, McMaster University, Hamilton, ON, Canada; Department of Medical Oncology, Nepean Cancer Care Centre, Kingswood, NSW, Australia; Department of Medical Oncology, Nepean Cancer Care Centre, Kingswood, NSW, Australia; NHMRC Clinical trials Centre, University of Sydney, Camperdown, NSW, Australia; Sydney Medical School, University of Sydney, Camperdown, NSW, Australia; Department of Medical Oncology, Nepean Cancer Care Centre, Kingswood, NSW, Australia; Sydney Medical School, University of Sydney, Camperdown, NSW, Australia; The Crown Princess Mary Cancer Centre, Westmead Hospital, Westmead, NSW, Australia

**Keywords:** melanoma, advanced cancer, metastatic cancer, prognosis, overall survival, life expectancy

## Abstract

**Background:**

We aim to provide survival scenario estimates for patients with advanced melanoma starting targeted therapies and immunotherapies.

**Materials and Methods:**

We sought randomized trials of targeted therapies and immunotherapies for advanced melanoma and recorded the following percentiles (represented survival scenario) from each overall survival (OS) curve: 90th (worst-case), 75th (lower-typical), 50th (median), 25th (upper-typical), and 10th (best-case). We tested whether these scenarios can be estimated for each OS curve by multiplying its median by 4 multiples: 0.25 (worst-case), 0.5 (lower-typical), 2 (upper-typical), and 3 (best-case).

**Results:**

We identified 15 trials with 8025 patients. For first-line combination targeted therapy treatment groups, the median (interquartile range, IQR) in months for each percentile was: 90th, 6.2 (6.0-6.5); 75th, 11.3 (11.3-11.4); and median, 24.4 (23.5-25.3). For the first-line combination immunotherapy treatment group, the percentiles in months were: 90th, 3.9 (2.8-4.5); 75th, 13.4 (10.1-15.4), median 73 (not applicable). In targeted therapy groups, simple multiples of the median OS were accurate for estimating the 90th percentile in 80%; 75th percentile in 40%; 25th percentile in 100%. In immunotherapy groups, these multiples were accurate at 0% for the 90th percentile, and 43% for the 75th percentile. The 90th percentile (worst-case scenario) was better estimated as 1/6× median OS, and the 75th percentile (lower-typical) as 1/3× median OS.

**Conclusions:**

Simple multiples of the median OS are a useful framework to estimate scenarios for survival for patients receiving targeted therapies, not immunotherapy. Longer follow-up is required to estimate upper-typical and best-case scenarios.

## Teaser

This study aimed to determine whether a novel method of estimating scenarios for survival (simple multiples of median overall survival) could be applied to patients with advanced melanoma receiving targeted therapy or immunotherapy, in order to provide clinicians with a framework to estimate and explain survival time to these patients.

**Implications for Practice**

Using simple multiples of the median OS to estimate worst-case and typical scenarios for survival remains a helpful framework to explain prognosis to patients with advanced melanoma treated with targeted therapies. It is possible that our simple multiples method may more accurately estimate the prognosis for patients being treated with immunotherapy for other tumor types. Therefore, there is an opportunity to test these multiples for trials of immunotherapy among other malignancies. For patients with advanced melanoma receiving immunotherapy, survival time may be best described as the percentage of patients alive at 1, 2, and 5 years. There is insufficient data on the longest-surviving trial participants. Extended follow-up is required to better understand long-term survival outcomes.

## Introduction

Most people with advanced cancer want information about their prognosis.^1,2^ Accurate information on expected survival time helps patients and doctors make treatment decisions, plan for the future, and prepare for the end of life.^3,4^

Unfortunately, many patients have a limited understanding of their prognosis, often overestimating likely survival time.^4,5^ Inadequate communication between oncologists and patients contributes to this misunderstanding.^6^ Many oncologists find it difficult to estimate and explain survival time in a way that is accurate yet conveys hope.^2^

**Figure 1.**
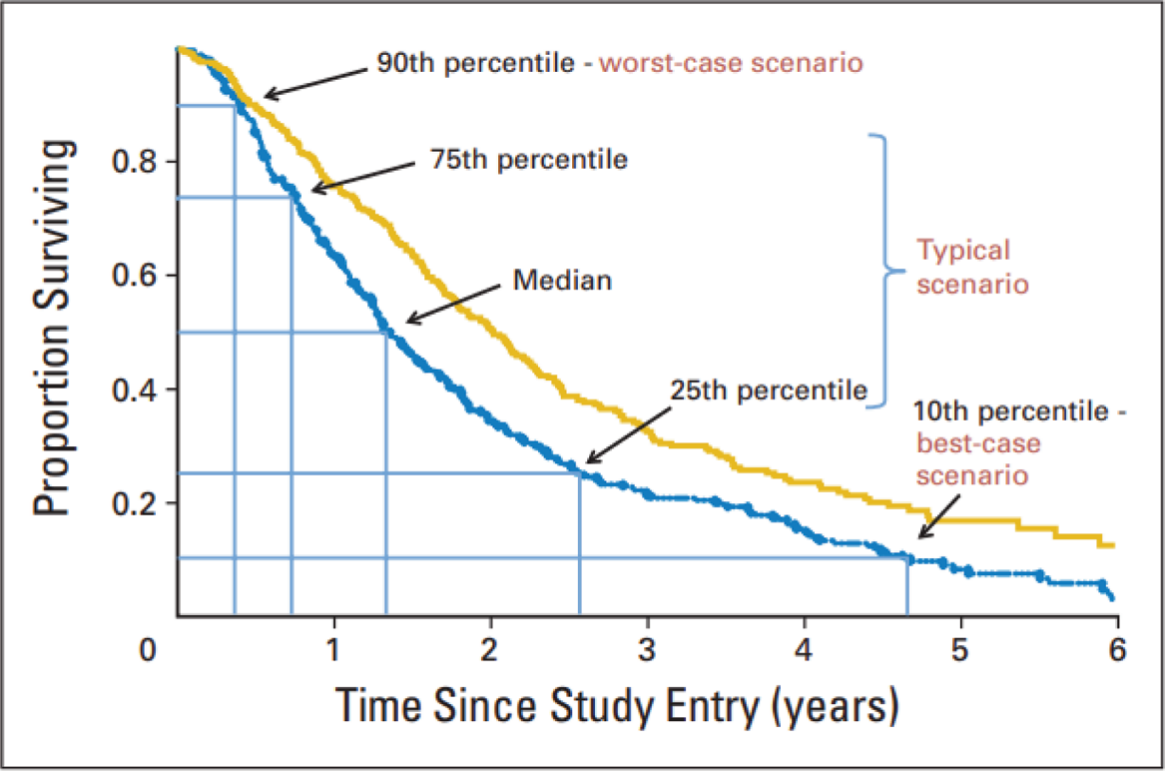
Survival curve percentiles and their corresponding scenarios.

We have previously proposed that worst-case, typical, and best-case scenarios are a useful framework to communicate expected survival time to patients with advanced cancer, and are preferable to providing a single-point estimate of the median survival.^5,7-10^ We have shown that the percentiles of an overall survival (OS) curve can be used to define ranges representing these scenarios.^11^ The 90th percentile, which represents the time at which 90% of the study cohort are still alive and 10% have died, can be used to represent the upper limit of the range for a worst-case scenario (shortest 10% of survival times); the interval between the 75th and 25th percentiles (middle 50%) can represent a range for the typical scenario and; the 10th percentile, where 10% are alive and 90% have died, can represent the lower limit of a range for the best-case scenario (longest 10%; Figure 1). We have also shown that simple multiples (0.25, 0.5, 2, and 3) of an OS curve’s median can be used to accurately estimate these percentiles in clinical trials of patients with advanced cancers treated with chemotherapy^5,12^ and targeted therapy.^9,10^

Historically, advanced melanoma had a poor prognosis. Recently, treatment has changed dramatically with the advent of targeted and immunotherapies. Combining targeted therapies dabrafenib and trametinib in first-line BRAF mutant advanced melanoma has shown 5-year survival of 34%.^13^ Similarly, single-agent first-line pembrolizumab has shown a 5-year survival of 38%.^13^ Estimating survival for patients with advanced melanoma has become challenging as these therapies improve survival compared to their chemotherapeutic predecessors. Rather than presenting a single-point estimate of the median OS or likelihood of surviving 5 years, providing ranges for typical, best-case, and worst-case scenarios can convey expected survival to patients in a more realistic and meaningful way.

We aimed to determine whether our method of estimating scenarios for survival can be applied to patients with advanced melanoma receiving targeted therapies and immunotherapies, providing clinicians with a framework to estimate and explain survival time to these patients.

## Materials and methods

We searched Medline, EMBASE, and the Cochrane Central Register of Controlled Trials for phase II or III randomized controlled trials of immunotherapy or targeted therapy for patients with advanced (unresectable stage IIIC and stage IV) melanoma.

We included trials published from January 2001 to June 2023. Trials were only included if there were greater than 90 patients in each treatment arm and a Kaplan-Meier curve for OS. Immunotherapy or targeted therapy must have been given in at least one arm of the trial and not in combination with chemotherapy. Trial arms were only included if they were consistent with current standards of care. The inclusion criteria and search terms are summarized in Supplementary Tables S1 and S2. For each trial, we recorded the year of publication, number of treatment arms, line of treatment, and median age. For each treatment arm, we recorded the treatments used, median OS, median progression-free survival (PFS), ECOG performance status, serum lactate dehydrogenase (LDH), V600 mutation status, and stage (unresectable stage III, stage IV, and M0-M1c disease).

Two authors independently traced each OS curve using the UN-SCAN-IT graph digitizing software (Silk Scientific, Orem, UT).^11^ The median and following percentiles (represented scenarios) were extracted from each curve: 90th (worst-case), 75th (lower-typical), 25th (upper-typical), and 10th (best-case). Discrepancies between the 2 measurements were resolved by repeated measurement.

Based on our previous work, we hypothesized that simple multiples of the median of each OS curve would approximate its percentiles representing the ranges for 3 scenarios as follows: 0.25 times the median for the 90th percentile (upper limit of worst-case scenario), 0.5 times for the 75th (lower limit of typical scenario), 2 times for the 25th (upper limit of typical scenario), and 3 times for the 10th (lower limit of the best-case scenario). For consistency with our previous work, we classified each estimate as “accurate” if it was within 0.66 to 1.33 times the actual value from the OS curve.

We assessed associations between survival and baseline characteristics in each trial arm by calculating Pearson’s correlation coefficients between the median survival and the summary measures of the baseline characteristics from each trial. These measures included ECOG performance status, age (on a continuous scale), LDH, sites of disease, and V600 mutation.

We first tested the accuracy of the scenarios in targeted therapies and immunotherapies regardless of the line of treatment. We then stratified the trial arms into first-line (>85% of patients receiving initial treatment) versus second- and subsequent-line (≤85% receiving initial treatment) for advanced disease.^14-30^ Arms were further grouped into single-agent versus combination therapy: first-line single-agent immunotherapy^18,31-35^ (Group 1), first-line combination immunotherapy^32,34-37^ (Group 2), first-line combination targeted therapy^13,38^ (Group 3), second-line immunotherapy^14-23^ (Group 4), and second-line targeted therapy^24-30^ (Group 5). Data regarding treatment that is no longer standard of care, including first-line single-agent targeted therapy, and single-agent tremelimumab, were extracted but not included in this manuscript.

## Results

### Trial characteristics

**Figure 2.**
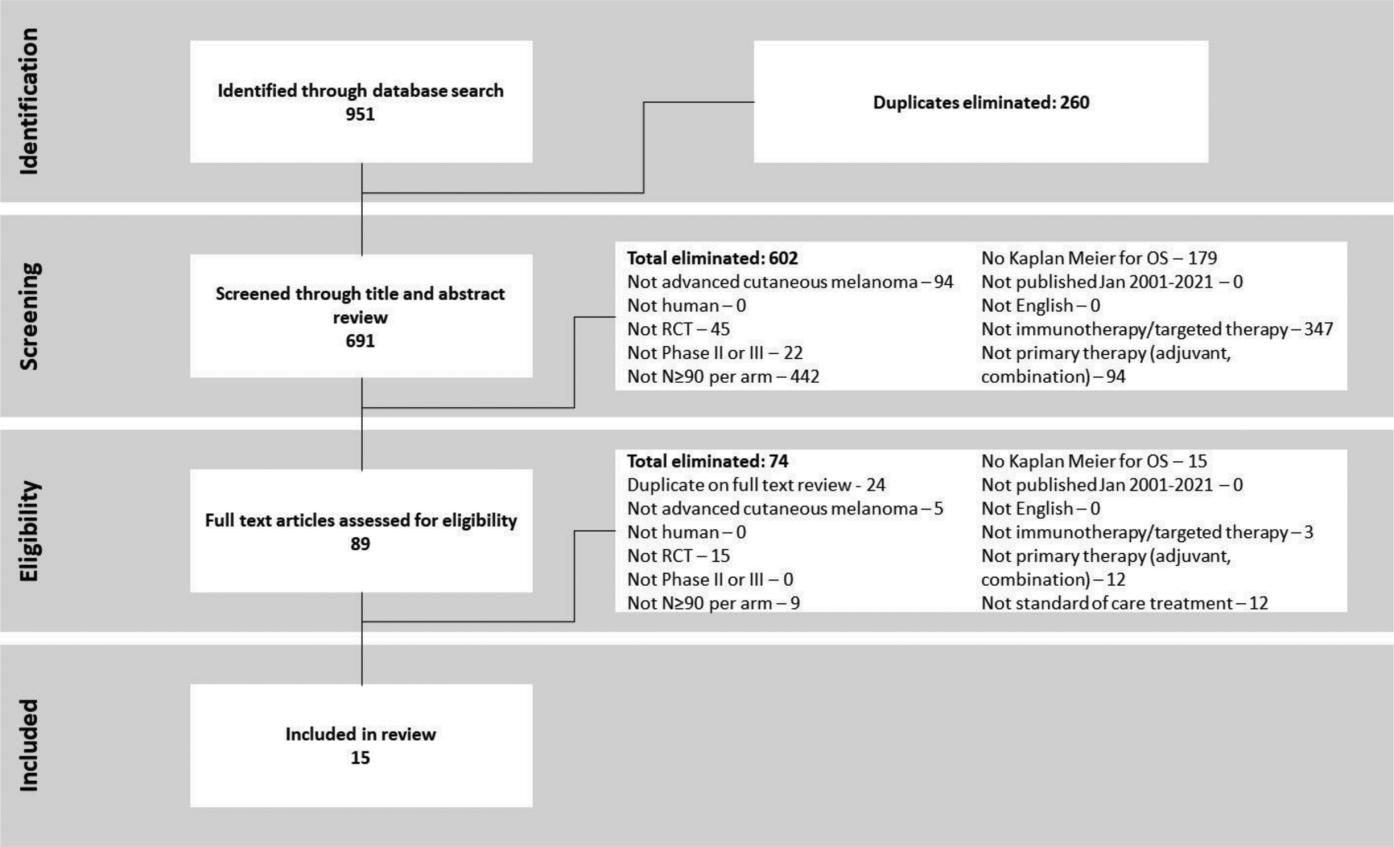
Trial selection flowchart.

Our search yielded 951 references from which 15 trials were included. The most common reasons for elimination were small sample size (≤90), interventions that were not immunotherapy or targeted therapy, and the absence of a Kaplan-Meier OS curve (Figure 2). The 15 trials included 8025 patients with 21 treatment arms (Table 1; Supplementary Tables S3 and S4). Median follow-up ranged from 9 to 58 months with a median of 18 months.

**Table 1.** Characteristics of included studies.

Reference	Phase	Total patient for OS	Number of arms	Intervention type (regimen)	First line (%)	Second line (%)+	ECOG 0-1 (%)	Median age^a^ (years)	Hazard ratio (95% CI)^a^	Median follow-up (months)^a^
Immunotherapy										
Ascierto et al^31^ Checkmate 066 NCT01721772	3	418	2	Nivolumab vs dacarbazine	100	0	100	64	0.46 (0.36-0.59), *P* < .001. (favors nivolumab vs dacarbazine)	Min 38.4
Hamid et al^17^ KEYNOTE-002 NCT01704287	2	540	3	Pembro 2 mg/kg vs pembro 10 mg/kg vs chemotherapy	0	100	99	60 (10 mg/kg) 62 (2 mg/kg)	0.74 (0.57-0.96) *P* = .0106 (favors pembrolizumab 10 mg/kg vs chemotherapy) 0.86 (0.67-1.10) *P* = .1173 (favors pembrolizumab 2 mg/kg vs chemotherapy)	17
Hodi et al^36^ CheckMate 069 NCT01927419	2	142	2	Nivolumab + ipilimumab vs ipilimumab	0	100	100	64	0.74 (0.43-1.26) *P* = 0.26 (favors nivolumab + ipilimumab vs ipilimumab)	24.5
Hodi et al^35^ Checkmate 067 NCT04540705.	3	945	3	Nivolumab + ipilimumab vs nivolumab vs ipilimumab	100	0	100	62 (ipilimumab) 60 (nivolumab) 61 (ipilimumab + nivolumab)	Not reported	Min 90
Larkin et al^14^ Checkmate 037 NCT01721746	3	272	2	Nivolumab vs. chemotherapy	0	100	100	59	0.95 (0.73-1.24) (nivolumab vs chemotherapy)	24
Lebbe et al^37^ CheckMate 511 NCT02714218	3 and 4	360	2	Nivolumab 1 mg/kg + ipilimumab 3 mg/kg vs nivolumab 3 mg/kg + ipilimumab 1 mg/kg	100	0	100	58 (nivolumab 1 mg/kg + ipilimumab 3 mg/kg) 58 (nivolumab 3 mg/kg + ipilimumab 1 mg/kg)	1.09 (0.73-1.62) (nivolumab 3 mg/kg + ipilimumab 1 mg/kg vs nivolumab 1 mg/kg + ipilimumab 3 mg/kg)	18.6 (nivolumab 1 mg/kg + ipilimumab 3 mg/kg) 18.8 (nivolumab 3 mg/kg + ipilimumab 1 mg/kg)
Long et al^16^ ECHO-301/KEYNOTE-252 NCT02752074	3	706	2	Epacadostat + pembrolizumab vs pembrolizumab	88	12	100	64 (epacadostat + pembrolizumab) 63 (pembrolizumab)	1.13(0.86-1.49) (epacadostat + pembrolizumab vs pembrolizumab + placebo)	12.4
Nathan et al^15^ Checkmate 172 NCT02156804	2	1008	1	Nivolumab	0	100	93	61	Not reported	16.8
Robert et al^18^ KEYNOTE 006 NCT01866319	3	834	3	Pembrolizumab vs ipilimumab	58	42	100	62 (combined pembrolizumab) 62 (combined ipilimumab)	0.73 (0.61-0.88), *P* = 00049 (favors pembrolizumab vs ipilimumab) 0.73 (0.57-0.92), *P* = .0036 (favors first-line pembrolizumab vs ipilimumab)	57.7
Tawbi et al^34^ RELATIVITY-047 NCT03470922	2 and 3	714	2	Relatlimab + nivolumab vs nivolumab	100	0	100	62 (nivolumab) 63 (nivolumab + relatlimab)	0.81 (0.64-1.01) (nivolumab + relatlimab vs nivolumab)	Min 13.2
Wolchok 2022 NCT01844505 Checkmate 067	3	945	3	Nivolumab + ipilimumab then nivolumab vs nivolumab vs ipilimumab	100	0	100	61% <65 39% 65+	0.52 (0.43-0.64) (nivolumab + ipilimumab) 0.63 (0.52-0.76) (nivolumab) NA (ipilimumab)	Min 77
Targeted therapy										
Algazi et al^27^ S1320 NCT02196181	2	206	2	Continuous dabrafenib + trametinib vs intermittent dabrafenib + trametinib	70	30	100^b^	61	1.03 (80% Cl 0.78–1.33), *P* = .93 (continuous vs intermittent)	24
Ascierto et al^25^ COLUMBUS NCT01909453	3	577	3	Combo (encorafenib + binimetinib) vs encorafenib or vemurafenib	^c^	^c^	100%	57 (combo) 54 (encorafenib) 56 (vemurafenib)	0.61(0.48-0.79) (favors combo vs vemurafenib)	48.80
Ascierto et al^38^ COBRIM NCT01689519	3	495	2	Cobimetinib + vemurafenib vs vemurafenib + placebo	100	0	100%	55 (vemurafenib + placebo) 56 (vemurafenib + cobimetinib)	0.8 (0.64-0.99) (favors cobimetinib + vemurafenib vs placebo + vemurafenib)	16.6 (vemurafenib + placebo) 21.2 (vemurafenib + cobimetinib)
Robert et al^13^ COMBI-d NCT01584648 COMBI-v NCT01597908	3	563	2	Dabrafenib + trametinib	100	0	73	55	Not reported	22

^a^Arms are labeled where more than one immunotherapy or targeted therapy arm is included. Where unlabeled, data refers to the sole immunotherapy or targeted therapy arm included in the study (eg, not chemotherapy).

^b^All ECOG 0-2, further breakdown not specified by authors.

^c^All patients were either untreated or progressed on first-line treatment.

Eleven trials included immunotherapy (ipilimumab, nivolumab, pembrolizumab, and relatlimab) and 4 included targeted therapy (cobimetinib, dabrafenib, encorafenib, trametinib, and vemurafenib). Most patients had an ECOG performance status of 0 or 1.

Six of 21 (28%) treatment arms had not reached median OS at the time of reporting. Follow-up was sufficient for the 25th percentile in only one of the treatment arms. None of the treatment arms met the 10th percentile. All 5 targeted therapy arms met the median OS, which allowed for the calculation of the multiples. Across all 16 immunotherapy arms, only 10 curves met the median OS.

### Survival outcomes

The mean values for the survival times of interest (as extracted from the survival curves) for each treatment group are shown in Table 2.

**Table 2.** Summary of median overall survival, progression-free survival and estimated scenarios for survival by treatment group.

	Group 1 (1L mono-immunotherapy)	Group 2 (1L combo-immunotherapy)	Group 3 (1L combo-targeted therapy)	Group 4 (2L immunotherapy)	Group 5 (2L targeted therapy)
*n* arms	6	5	2	5	3
*n* arms reaching median OS	4	1	2	5	3
Mean (IQR) in months					
Worst-case (90th percentile)	4.4 (3.9-4.9)	3.9 (2.8-4.5)	6.2 (6-6.5)	2.7 (2.2-2.7)	5.9 (5-6.6)
Lower-typical (75th percentile)	10.9 (10.5-11.4)	13.4 (10.1-15.4)	11.3 (11.3-11.4)	7.2 (6.2-7.3)	11.3 (10.9-11.6)
Median OS	36.5 (36-38)	73 (NA)	24.4 (23.5-25.3)	20.6 (14.6-24.7)	30.7 (29.2-31.4)
Upper-typical (25th percentile)	NR (NR-NR)	NR (NR-NR)	NR (NR-NR)	32.3 (32.3-32.3)	NR (NR-NR)
Best-case (10th percentile)	NR (NR-NR)	NR (NR-NR)	NR (NR-NR)	NR (NR-NR)	NR (NR-NR)
Median PFS	4.8 (2.9-11.6)	10.1 (9.7-10.5)	11.9 (11.5-12.2)	3.0 (2.8-8.4)	7.1 (5.1-8.6)
Mean % (IQR) [number of treatment arms assessable]					
1-year survival	73 (73-74) [*n* = 6]	77 (73-80) [*n* = 5]	74 (74-74) [*n* = 2]	61 (56-66) [*n* = 5]	72 (70-73) [*n* = 3]
2-year survival	58 (58-58) [*n* = 4]	64 (63-65) [*n* = 4]	51 (50%-52%) [*n* = 2]	44 (38-51) [*n* = 5]	57 (56-57) [*n* = 3]
5-year survival	43 (43-44) [*n* = 2]	51 (NA) [*n* = 1]	33 (32-34) [*n* = 2]	40 (40-40) [*n* = 1]	NR (NR-NR) [*n* = 0]

#### Scenarios for survival in targeted therapy groups

In the 2 treatment arms of first-line combination targeted therapy (Group 3), the mean (IQR) for the worst-case scenario was 6.2 months (6-6.5); lower-typical scenario was 11.3 months (11.3-11.4); and median OS was 24.4 months (23.5-25.3). Upper-typical and best-case scenarios were not reached in any treatment arms. Mean 1-year and 2-year survival rates were 74% (74-74%) and 51% (50-52%), respectively.

In the 3 treatment arms of second- and subsequent-line targeted therapy (Group 5), the mean (IQR) for the worst-case scenario was 5.9 months (5-6.6); lower-typical scenario was 8.9 months (6.5-10.9); median OS was 20.5 months (15.9-27.8); upper-typical was 33.2 months (19.4-46.6); and best-case scenario was 25.7 months (24.9-26.4). Mean 1-year and 2-year survival rates were 63% (IQR 59-71%) and 41% (32-56%), respectively.

#### Scenarios for survival in immunotherapy groups

In the 6 first-line single-agent immunotherapy treatment arms (Group 1),^18,31-35^ the mean (IQR) of the worst-case scenario was 4.4 months (3.9-4.9); lower-typical was 10.9 months (10.5-11.4); median OS was 36.5 months (36-38). The upper-typical (25th percentile) and best-case scenarios (10th percentile) were not reached in any curves. The mean (IQR) 1-year and 2-year survival rates were 73% (73%-74%) and 58% (43%-44%), respectively.

In the 5 first-line combination immunotherapy treatment arms (Group 2), the mean (IQR) in months for the worst-case scenario was 3.9 (2.8-4.5); lower-typical was 13.4 (10.1-15.4); median OS was 73 (NA). The upper-typical and best-case scenarios were not reached in any curves. The mean 1-year and 2-year survival rates were 77% (73-80%) and 64 (63-65%), respectively.

In the 5 second- and subsequent-line immunotherapy treatment arms (Group 4), the mean (IQR) in months for the worst-case scenario was 2.7 (2.2-2.7); lower-typical scenario 7.2 (6.2-7.3); the median OS was 20.6 (14.6-24.7); upper-typical was 32.3 (32.3-32.3). The best-case scenario was not reached. Mean 1-year and 2-year survival rates were 61% (IQR 56-66%) and 44% (38-51%), respectively.

#### Accuracy of survival scenarios

The proportion of curves where simple multiples were accurate for estimating scenarios is outlined in Table 3. Across both targeted therapy treatment groups (Groups 3 and 5), simple multiples of the median OS accurately estimated the worst-case scenario in 80% (4 of 5 treatment arms that met the median OS), and the lower-typical scenario in 40% (2 of 5 treatment arms). Accuracy for estimating the upper-typical scenario and best-case scenario could not be determined because the follow-up was too short. The simple multiples method overestimated the actual worst-case and lower-typical scenarios, meaning that the proportions of participants who died early in these trials were higher than we predicted (1 worst-case estimate and 3 lower-typical estimates were overestimated by our multiples).

**Table 3.** Proportions of OS curves for each treatment group where simple multiples were accurate for estimating scenarios (*n* = number of OS treatment arms that met the percentile).

	Group 1 (1L mono-immunotherapy)	Group 2 (1L combo-immunotherapy)	Group 3 (1L combo-targeted therapy)	Group 4 (2L all immunotherapy)	Group 5 (2L all targeted therapy)
0.25× median est. 90th percentile (worst-case)	0% (*n* = 4)	0% (*n* = 1)	100% (*n* = 2)	0% (*n* = 5)	66.7% (*n* = 3)
0.5× median est. 75% percentile (lower-typical)	0% (*n* = 4)	0% (*n* = 1)	100% (*n* = 2)	60% (*n* = 5)	0% (*n* = 3)
2× median est. 25% percentile (upper-typical)	NR (*n* = 0)	NR (*n* = 0)	100% (*n* = 2)	100% (*n* = 1)	NR (*n* = 0)
3× median est. 10th percentile (best-case)	NR (*n* = 0)	NR (*n* = 0)	NR (*n* = 0)	NR (*n* = 0)	NR (*n* = 0)

Across all 3 immunotherapy treatment groups (Groups 1, 2, and 4), simple multiples accurately estimated the worst-case scenario in 0% (0 of 10 treatment arms that met the median OS), the lower-typical scenario in 30% (3 of 10 treatment arms), and the upper-typical scenario in 100% (1 treatment arm). Follow-up was too short to evaluate the best-case scenario. Our multiples overestimated the worst-case and lower-typical scenarios in 100% of estimates.

Among immunotherapy treatment groups, the worst-case scenario was more accurately estimated using 1/6× the median OS which was accurate in 60% of treatment groups. The lower-typical scenario was more accurately estimated using the multiple 1/3× the median OS, which was accurate in 90% of treatment arms.

#### Distribution of survival and characteristics correlated with survival

**Figure 3.**
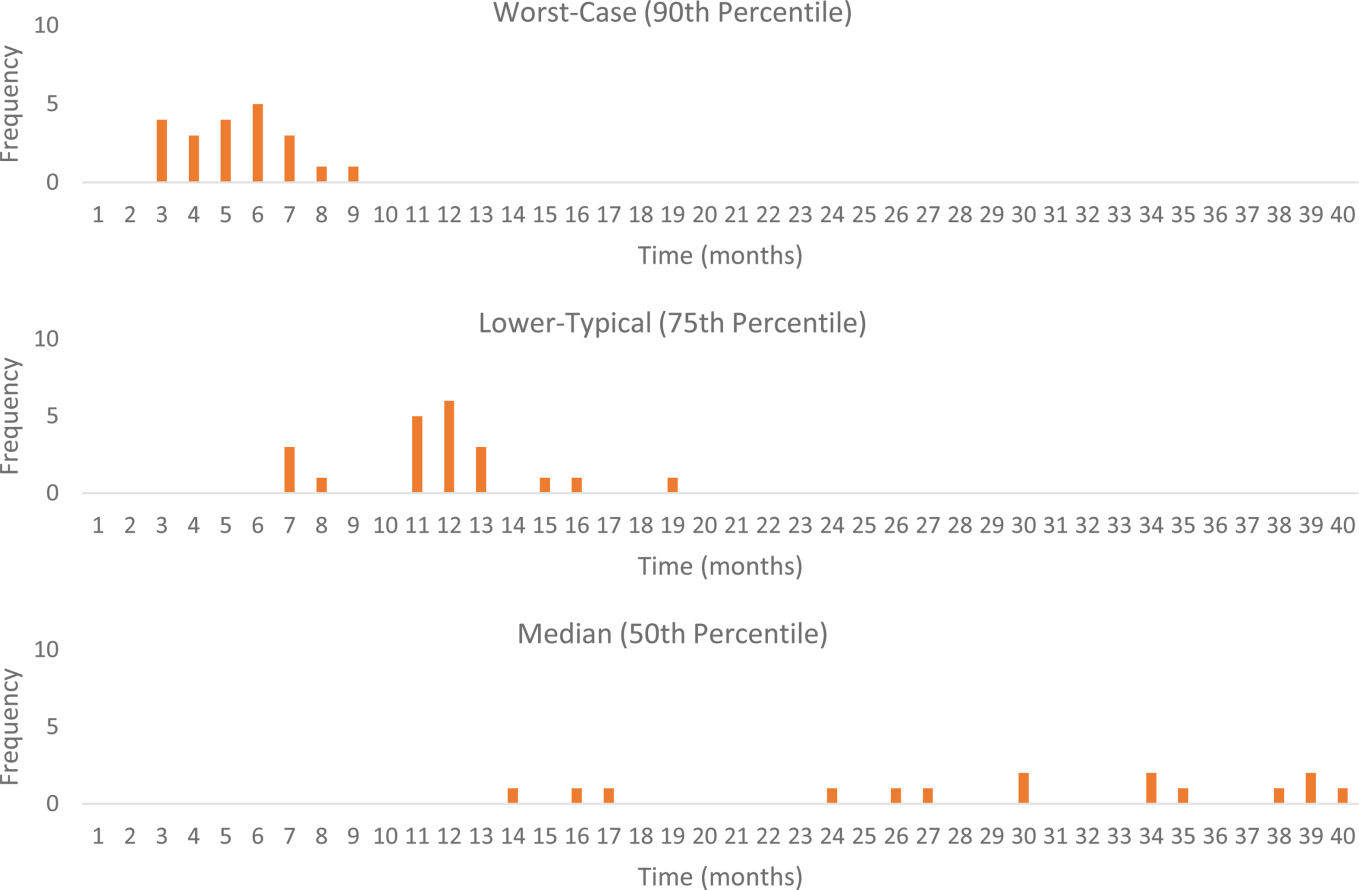
Distribution of scenarios for survival.

The distribution of survival times for each of the percentiles (scenarios) is shown in Figure 3. Each was skewed toward longer survival times. The range of the distribution broadened as the scenarios increased from worst-case to best-case scenario.

Supplementary Table S5 summarizes the correlation between the characteristics of a trial and its median OS, calculated with the Pearson correlation. Longer median OS was significantly associated with trials where there was a higher percentage of participants with grade 3+ adverse events. There were insufficient data to perform a reliable multivariable analysis.

## Discussion

Targeted therapies and immunotherapies have greatly improved survival for patients with advanced melanoma, with the median OS for first-line combination immunotherapy being over 6 years.^35^ Simple multiples of the median OS accurately estimated the worst-case (90th percentile), and lower-typical (75th percentile) scenarios in the targeted therapy treatment groups but were inaccurate in the immunotherapy groups where they underestimated the frequency of early deaths. Few trials had follow-up beyond the median OS (only one treatment arm reached the 25th percentile and none reached the 10th percentile) and therefore we have minimal information on long-term outcomes for these patients.

The targeted therapy results are similar to the findings in our previous work in metastatic HER2-positive breast cancer treated with dual HER2-targeted therapies,^9^ where the simple multiples method was accurate for the short-term outcomes but follow-up was insufficient to quantify the best-case scenario.

The survival curves for targeted therapy in advanced melanoma follow a similar shape to the survival curves for chemotherapy^5,10^ and other targeted therapy trials.^9^ Therefore, the same simple multiples that accurately estimated survival in previous studies of chemotherapy and targeted therapy were similarly accurate here. Assuming that the same shape is followed beyond the median, we hypothesize that the best-case scenario for patients being treated with targeted therapy for advanced melanoma can be estimated as longer than 3 times the median OS.

For example, to estimate survival times for a patient starting combination targeted therapies, a median OS time of 24 months is a useful starting point. The 3 scenarios could then be calculated as a worst-case scenario of less than 6 months (<0.25 times 24 months), a typical scenario of 12-48 months (0.5-2 times 24 months), and a best-case scenario of more than 6 years (>3 times 24 months). These scenarios for survival can then be explained to a patient as: “If we imagine 100 people in the same situation, then we would expect the 5-10 who did worst would die within 6 months; the middle 50 would live for 1-4 years, and the 5-10 who did best would live longer than 6 years.” Patients should be informed that the best-case scenario remains uncertain due to minimal long-term follow-up of the clinical trials.

There is limited long-term survival information available for patients with advanced melanoma receiving first-line combination immunotherapy. To date, only one treatment arm (Checkmate 067^35^) of combination immunotherapy has reached the median OS and 4 of 6 treatment arms of single-agent immunotherapy have reached the median OS. With this limitation, we found that our simple multiples of the median did not accurately estimate the worst-case (90th percentile) or lower-typical scenarios (75th percentile). Across all 16 immunotherapy arms (Groups 1, 2, and 4), multiples of 1/4 and 1/2 the median, based on chemotherapy trials, tended to overestimate the worst-case and lower-typical scenarios, underestimating the number of early deaths for patients in these groups. These scenarios were more accurately estimated using 1/6× and 1/3× the median OS. For patients being treated with immunotherapy, the worst-case and lower-typical scenarios may be better estimated and explained as 1/6 and 1/3 of the median OS. Follow-up duration was insufficient to calculate the upper-typical and best-case scenarios in this group.

The shape of these OS curves from trials of immunotherapy for melanoma differed subtly from the shape of those in trials of chemotherapy and targeted therapies. Higher proportions of participants died earlier than 1/4 of the median and 1/2 the median in trials of immunotherapy for melanoma than in trials of chemotherapy or targeted therapies, meaning that the predicted worst-case and lower-typical scenarios should be shorter. Objective tumor responses and survival may be more durable with immunotherapy than with chemotherapy or targeted therapies, and often extended beyond the maximum reported follow-up in these trials. In addition, patients are living longer, and trial follow-up is not yet in-line with this increase in survival. It may be more accurate and helpful to estimate and explain survival time to these patients using percentages expected to be alive at 1, 2, and 5 years.

This paper provides a framework for clinicians to estimate and explain survival times in patients with advanced melanoma receiving targeted therapies. There has been a paucity of data on how to communicate survival to patients treated with targeted and immunotherapies, whereby previous estimates of survival have relied on trials of chemotherapy, which are now not relevant in the management of advanced melanoma. This study is the most comprehensive summary, to date, of prognostic information in patients with advanced melanoma receiving targeted and immunotherapies.

One of the main limitations of this study is that follow-up was insufficient in most trials to accurately obtain the long-term outcomes, especially the best-case scenario, as trials were published either before, at, or soon after, the median OS was reached. This limits what clinicians can tell their patients about the potential best-case scenario. The small number of trials included in this paper was quite heterogenous, particularly in the combination immunotherapy group. This limits our ability to make conclusions that encompass all trials within the group. Another limitation is that the survival times reported in this review are of a clinical trial population, who are generally fitter than a real-world cohort.

Given the method of using 0.25×, 0.5×, 2×, and 3× the median was not accurate for estimating scenarios for survival for patients receiving immunotherapy, further research in patients with melanoma and other advanced cancers receiving immunotherapy is needed. Different multiples may be more accurate, such as 1/6 and 1/3, or a different approach entirely. It is important to ask patients in this situation for their preferences regarding prognostic information, in particular quantitative information.

## Supplementary material

Supplementary material is available at *The Oncologist* online.

oyae089_suppl_Supplementary_Material

## Data Availability

No new data were generated or analyzed in support of this research.

## References

[1] Kiely B, McCaughan G, Christodoulou S, et al Using scenarios to explain life expectancy in advanced cancer: attitudes of people with a cancer experience. Support Care Cancer. 2013;21(2):369-376. 10.1007/s00520-012-1526-4 22717918

[2] Hagerty RG, Butow P, Ellis P, et al Cancer patient preferences for communication of prognosis in the metastatic setting. J Clin Oncol. 2004;22(9):1721-1730. 10.1200/JCO.2004.04.095 15117995

[3] Clayton JM, Butow PN, Arnold RM, Tattersall MHN. Discussing end-of-life issues with terminally ill cancer patients and their carers: a qualitative study. Support Care Cancer. 2005;13(8):589-599. 10.1007/s00520-004-0759-2 15645187

[4] Enzinger AC, Zhang B, Schrag D, Prigerson HG. Outcomes of prognostic disclosure: associations with prognostic understanding, distress, and relationship with physician among patients with advanced cancer. J Clin Oncol. 2015;33(32):3809-3816. 10.1200/JCO.2015.61.9239 26438121 PMC4737862

[5] Kiely BE, Soon YY, Tattersall MH, Stockler MR. How long have I got? Estimating typical, best-case, and worst-case scenarios for patients starting first-line chemotherapy for metastatic breast cancer: a systematic review of recent randomized trials. J Clin Oncol. 2011;29(4):456-463. 10.1200/JCO.2010.30.2174 21189397

[6] Anderson WG, Chase R, Pantilat SZ, Tulsky JA, Auerbach AD. Code status discussions between attending hospitalist physicians and medical patients at hospital admission. J Gen Intern Med. 2011;26(4):359-366. 10.1007/s11606-010-1568-6 21104036 PMC3055965

[7] Stockler MR, Tattersall MH, Boyer MJ, et al Disarming the guarded prognosis: predicting survival in newly referred patients with incurable cancer. Brit J Cancer. 2006;30(94):208-212. 10.1038/sj.bjc.6602908 PMC2361107 16404420

[8] Smith-Uffen ME, Johnson SB, Martin AJ, et al Estimating survival in advanced cancer: a comparison of estimates made by oncologists and patients. Support Care Cancer. 2020;28(7):3399-3407. 10.1007/s00520-019-05158-5 31781946

[9] Vasista A, Stockler MR, West TA, Wilcken N, Kiely BE. More than just the median: calculating survival times for patients with HER2 positive, metastatic breast cancer using data from recent randomised trials. Breast. 2017;31(1):99-104. 10.1016/j.breast.2016.10.007 27829202

[10] West TA, Kiely BE, Stockler MR. Estimating scenarios for survival time in men starting systemic therapies for castration-resistant prostate cancer: a systematic review of randomised trials. Eur J Cancer. 2014;50(11):1916-1924. 10.1016/j.ejca.2014.04.004 24825113

[11] Scientific S. UN-SCAN-IT Graph Digitizing Software. Silk Scientific Inc.; 1988-2018.

[12] Kiely B, Alam M, Blindman P, et al Estimating typical, best-case and worst-case life expectancy scenarios for patients starting chemotherapy for advanced non-small-cell lung cancer: a systematic review of contemporary randomized trials. Lung Cancer. 2012;77(3):537-544. 10.1016/j.lungcan.2012.04.017 22609149

[13] Robert C, Grob J, Stroyakovskiy D, et al Five-year outcomes with dabrafenib plus trametinib in metastatic melanoma. New Engl J Med. 2019;381(7):626-636. 10.1056/NEJMoa1904059 31166680

[14] Larkin J, Minor D, D’Angelo S, et al Overall survival in patients with advanced melanoma who received nivolumab versus investigator’s choice chemotherapy in Checkmate 037: a randomized, controlled, open-label phase iii trial. J Clin Oncol. 2018;36(4):383-390. 10.1200/JCO.2016.71.8023 28671856 PMC6804912

[15] Nathan P, Ascierto PA, Haanen J, et al Safety and efficacy of nivolumab in patients with rare melanoma subtypes who progressed on or after ipilimumab treatment: a single-arm, open-label, phase II study (CheckMate 172). Eur J Cancer. 2019;119:168-178. 10.1016/j.ejca.2019.07.010 31445199

[16] Long GV, Dummer R, Hamid O, et al Epacadostat plus pembrolizumab versus placebo plus pembrolizumab in patients with unresectable or metastatic melanoma (ECHO-301/KEYNOTE-252): a phase 3, randomised, double-blind study. Lancet Oncol. 2019;20(8):1083-1097. 10.1016/S1470-2045(19)30274-8 31221619

[17] Hamid O, Puzanov I, Dummer R, et al Final analysis of a randomised trial comparing pembrolizumab versus investigator-choice chemotherapy for ipilimumab-refractory advanced melanoma. Eur J Cancer. 2017;86:37-45. 10.1016/j.ejca.2017.07.022 28961465

[18] Robert C, Ribas A, Schachter J, et al Pembrolizumab versus ipilimumab in advanced melanoma (KEYNOTE-006): post-hoc 5-year results from an open-label, multicentre, randomised, controlled, phase 3 study. Lancet Oncol. 2019;20(9):1239-1251. 10.1016/S1470-2045(19)30388-2 31345627

[19] Lebbe C, Weber J, Maio M, et al Survival follow-up and ipilimumab retreatment of patients with advanced melanoma who received ipilimumab in prior phase II studies. Ann Oncol. 2014;25(11):2277-2284. 10.1093/annonc/mdu441 25210016 PMC4990834

[20] Hodi FS, Lee S, McDermott DF, et al Ipilimumab plus sargramostim vs ipilimumab alone for treatment of metastatic melanoma a randomized clinical trial. JAMA. 2014;312(17):1744-1753. 10.1001/jama.2014.13943 25369488 PMC4336189

[21] Ascierto PA, Del Vecchio M, Robert C, et al Ipilimumab 10 mg/kg versus ipilimumab 3 mg/kg in patients with unresectable or metastatic melanoma: a randomised, double-blind, multicentre, phase 3 trial. Lancet Oncol. 2017;18(5):611-622. 10.1016/S1470-2045(17)30231-0 28359784

[22] Hodi FS, O’Day SJ, McDermott DF, et al Improved survival with ipilimumab in patients with metastatic melanoma. New Engl J Med. 2010;363(8):711-723. 10.1056/NEJMoa1003466 20525992 PMC3549297

[23] Kirkwood JM, Lorigan P, Hersey P, et al Phase II trial of tremelimumab (cp-675,206) in patients with advanced refractory or relapsed melanoma. Clin Cancer Res. 2010;16(3):1042-1049. 10.1158/1078-0432.CCR-09-2033 20086001

[24] Robert C, Flaherty K, Nathan P, et al Five-year outcomes from a phase 3 METRIC study in patients with BRAF V600 E/K mutant advanced or metastatic melanoma. Eur J Cancer. 2019;109:61-69. 10.1016/j.ejca.2018.12.015 30690294

[25] Ascierto PA, Dummer R, Gogas HJ, et al Update on tolerability and overall survival in COLUMBUS: landmark analysis of a randomised phase 3 trial of encorafenib plus binimetinib vs vemurafenib or encorafenib in patients with BRAF V600emutant melanoma. Eur J Cancer. 2020;126:33-44. 10.1016/j.ejca.2019.11.016 31901705

[26] Hauschild A, Ascierto PA, Schadendorf D, et al Long-term outcomes in patients with BRAF V600-mutant metastatic melanoma receiving dabrafenib monotherapy: analysis from phase 2 and 3 clinical trials. Eur J Cancer. 2020;125:114-120. 10.1016/j.ejca.2019.10.033 31864178 PMC8073226

[27] Algazi AP, Othus M, Daud AI, et al Continuous versus intermittent BRAF and MEK inhibition in patients with BRAF-mutated melanoma: a randomized phase 2 trial. Nat Med. 2020;26(10):1564-1568. 10.1038/s41591-020-1060-8 33020646 PMC8063889

[28] McArthur G, Maio M, Arance A, et al Vemurafenib in metastatic melanoma patients with brain metastases: an open-label, single-arm, phase 2, multicentre study. Ann Oncol. 2017;28(3):634-641. 10.1093/annonc/mdw641 27993793

[29] Sosman JA, Kim KB, Schuchter L, et al Survival in BRAF V600–Mutant advanced melanoma treated with Vemurafenib. N Engl J Med. 2012;366(8):707-714. 10.1056/NEJMoa1112302 22356324 PMC3724515

[30] Dummer R, Schadendorf D, Ascierto PA, et al Binimetinib versus dacarbazine in patients with advanced NRAS-mutant melanoma (NEMO): a multicentre, open-label, randomised, phase 3 trial. Lancet Oncol. 2017;18(4):435-445. 10.1016/S1470-2045(17)30180-8 28284557

[31] Ascierto PA, Long GV, Robert C, et al Survival outcomes in patients with previously untreated BRAF wild-type advanced melanoma treated with nivolumab therapy. JAMA. 2018;5(2):187-194. 10.1001/jamaoncol.2018.4514 PMC6439558 30422243

[32] Regan MM, Mantia CM, Werner L, et al Treatment-free survival over extended follow-up of patients with advanced melanoma treated with immune checkpoint inhibitors in CheckMate 067. J ImmunoTher Cancer. 2021;9(11):e003743-e003748. 10.1136/jitc-2021-003743 34799400 PMC8606772

[33] Ribas A, Kefford R, Marshall MA, et al Phase III randomized clinical trial comparing tremelimumab with standard-of-care chemotherapy in patients with advanced melanoma. J Clin Oncol. 2013;31(5):616-622. 10.1200/JCO.2012.44.6112 23295794 PMC4878048

[34] Tawbi J, Hodi S, Lipson E, et al. Nivolumab (NIVO) + relatlimab (RELA) versus NIVO in previously untreated metastatic or unresectable melanoma: OS and ORR by key subgroups from RELATIVITY-047. Paper presented at: ASCO; 2022; Chicago, IL.

[35] Hodi S, Chiarion-Sileni V, Lewis K. Long-term survival in advanced melanoma for patients treated with nivolumab plus ipilimumab in CheckMate 067. Paper presented at: ASCO; 2022; Chicago, IL.

[36] Hodi FS, Chesney J, Pavlick AC, et al Combined nivolumab and ipilimumab versus ipilimumab alone in patients with advanced melanoma: 2-year overall survival outcomes in a multicentre, randomised, controlled, phase 2 trial. Lancet Oncol. 2016;17(11):1558-1568. 10.1016/S1470-2045(16)30366-7 27622997 PMC5630525

[37] Lebbe C, Meyer N, Mortier L, et al Evaluation of two dosing regimens for nivolumab in combination with ipilimumab in patients with advanced melanoma: results from the phase IIIb/IV CheckMate 511 trial. J Clin Oncol. 2019;37(11):867-875. 10.1200/JCO.18.01998 30811280 PMC6455714

[38] Ascierto PA, Dreno B, Larkin J, et al 5-Year outcomes with cobimetinib plus vemurafenib in brafv600 mutation–positive advanced melanoma: extended follow-up of the coBRIM study. Clin Cancer Res. 2021;27(19):5225-5235. 10.1158/1078-0432.CCR-21-0809 34158360 PMC9401485

