# Supplementary material for: Estimating scenarios for survival time in patients with advanced melanoma receiving immunotherapy and targeted therapy
Source: Oncologist. 2024 May 20;29(11):922–30. doi: 10.1093/oncolo/oyae089 (PMC11546645; doi:10.1093/oncolo/oyae089)
Supplement: oyae089_suppl_Supplementary_Material [file oyae089_suppl_supplementary_material.docx]

Supplementary Material

| **Supplementary Table 1**. Eligibility criteria. | |
| --- | --- |
| Category | Eligibility criteria |
| Date of publication | 01/01/2001 – 28/06/2023 |
| Language of publication | English |
| Publication type | Phase II or III randomized controlled trials.  Excluded:   - Grey literature - Proceedings of conferences - Unpublished abstracts - PhD theses - Books - Professional guidelines - Non-original research (e.g. opinion pieces, commentaries, editorials, reviews) |
| Study characteristics | - N ≥ 90 patients per treatment arm - Must include Kaplan-Meier curve for overall survival |
| Intervention | - Immunotherapy or targeted therapy given in at least one arm as primary therapy (first or second line, not in combination with chemotherapy) |
| Population | Human, adult participants (18 years or older). |

| **Supplementary Table 2**. Key words used in literature search. | | | | |
| --- | --- | --- | --- | --- |
| Term type | Concept 1: Advanced cutaneous melanoma. | Concept 2: Immunotherapy. | Concept 3: Clinical trial. | Concept 4: Overall survival. |
| MeSH heading | Melanoma  Melano*  Naevocarcinom*  Nevocarcinom*  Neoplasm    AND^A^    Advanced*  Metastasis [or Neoplasm metastasis]  Metasta* | Antineoplastic Agents  Antibodies, Monoclonal  CTLA-4 Antigen  Immunotherapy  Ipilimumab  Nivolumab  Proto-Oncogene Proteins B-raf  Programmed Cell Death 1 Receptor  Therapeutics  Therapy  Treatment outcome  Vemurafenib | Controlled clinical trial  Clinical Trial  Double-Blind Method  Random Allocation  Randomized Controlled Trials as Topic  Single-Blind Method | Survival  Survival Analysis  Survival Rate |
| Key term |  | Agent*  Antineoplas*  Antibod*  Anti-pd-1  Anti-ctla-4  Binimetinib*  Cobimetinib*  Dabrafenib*  Drug*  Encorafenib*  Immunotherap*  Inhibitor*  Intervention*  Management*  Pembrolizumab*  Pharma*  Systemic*  Therap*  Treat*  Trametinib* | Assign*  Allocat*  Crossover*  Cross over*  Factorial*  Groups  Placebo*  Random*  Trial  Volunteer* | Surviv* |

Keywords within concepts were combined with OR. Concepts were combined with AND.

^A^ The keywords within Concept 1 were combined with AND.

| **Supplementary Table 3**. Characteristics of the 15 included trials. | | | | |
| --- | --- | --- | --- | --- |
| Characteristic |  | | | |
| Year of publication  *n* (%) | 2014  2015  2016  2017  2018 | 1 (6.7)  0 (0)  1 (6.7)  1 (6.7)  2 (13.3) | 2019  2020  2021  2022 | 5 (33.3)  2 (13.3)  1 (6.7)  2 (13.3) |
| Number of treatment groups compared n (%)  1  2  3 | 1 (6.7)  10 (66.7)  6 (26.7) | | | |
| Follow-up, months  Median (*n*), Range | 24 (10), 12-58 | | | |
| Number patients total analyzed for OS  Median (n), Range | 540 (15), 142-1008 | | | |
| Age, years  Median (n), Range | 63 (5), 59-64 | | | |
| Immunotherapy  Overall n (%) | 11 (73) | | | |
| Targeted therapy  Overall n (%) | 4 (27) | | | |
| ECOG 0-1  Median (*n*), Range | 100 (14), 73-100 | | | |
| ECOG 2+  Median (*n*), Range | 0 (14), 0-27 | | | |

| **Supplementary Table 4**. Characteristics of the 21 included treatment arms. | | | | | | |
| --- | --- | --- | --- | --- | --- | --- |
| Characteristic | Group 1  1L mono-immunotherapy (n=6) | Group 2  1L combo-immunotherapy (n=5) | Group 3  1L combo-targeted therapy (n=2) | Group 4  2L all immunotherapy (n=5) | Group 5  2L all targeted therapy  (n=3) |  |
| Sample size  Median (n), Range | 352 (5), 210-368 | 180 (5), 95-355 | 406 (2), 248-563 | 272 (5), 180-723 | 105 (3), 101-192 |  |
| Follow-up, months  Median (n*), Range | 35.1 (4), 12.4-90 | 18.8 (3), 18.6-24.5 | 22 (2), 21-22 | 28 (5), 16.8-57.7 | 24 (3), 24-48.8 |  |
| Age, years  Median (n), Range | 63 (5), 60-64 | 61 (5), 59-64 | 55 (2), 55-55 | 61 (5), 59-62 | 57 (1), NA |  |
| Female, %  Median (n), Range | 39.8 (5), 36.1-42.4 | 41 (5), 34-43 | 42 (2), 41-43 | 39.8 (5), 35-42.2 | 40.1 (3), 36-59 |  |
| Site of metastasis  M0-M1b %  Median (n), Range | 39 (4), 36-42 | 43 (5), 42-56 | NA (0), NA-NA | 25.8 (4), 17.7-34.8 | 45.4 (3), 35.9-45.7 |  |
| M0 %  Median (n), Range | 4.5 (2), 4-5 | 9 (2), 8-10 | 6 (2), 3-9 | 2.1 (4), 1.1-3.6 | 16.7 (3), 4.7-23.1 |  |
| M1a %  Median (n), Range | 11.5 (2), 11-12 | 31 (2), 16-45 | 15 (2), 13-16 | 9.6 (4), 4.4-13.8 | 31.6 (3), 13.5-41.2 |  |
| M1b %  Median (n), Range | 21.5 (2), 21-22 | 28 (1), NA | 18 (2), 16-19 | 14.8 (4), 9.4-18.9 | 44.4 (3), 17.7-50 |  |
| M1c %  Median (n), Range | 61 (4), 58-64 | 57 (5), 43-58 | 62 (2), 59-64 | 75 (5), 44.3-82.3 | 54.5 (3), 54.3-64.1 |  |
| Brain mets %  Median (n), Range | 4 (3), 2-5 | 2 (5), 0-4 | 5 (2), 1-9 | 9.2 (5), 0-20 | 0 (1), NA |  |
| LDH ≤ ULN %  Median (n), Range | 63.5 (4), 57.1-68 | 63 (5), 57-74) | 60 (2), 54-65 | 55.6 (5), 48-66.4 | 41.3 (3), 34.9-71 |  |
| LDH > ULN %  Median (n), Range | 36.3 (4), 32-38 | 36 (5), 26-43 | 40 (2), 34-46 | 43.3 (5), 32.2-52 | 55.3 (3), 29-61.5 |  |
| % V600 mut % Median (n), Range | 45 (3), 44-100 | 41 (5), 23-100 | 100 (1), NA | 24.4 (5), 22-63.8 | 100 (3), 100-100 |  |
| Grade 3+ AE %  Median (n), Range | 19.5 (4), 15-22 | 45 (5), 34-59 | 48 (2), 37-59 | 19 (5), 13.5-47 | 43 (3), 34-68.2 |  |

*number of arms that reported *median* follow-up

| **Supplementary Table 5.** Correlation between trial characteristic and median overall survival. | | | |
| --- | --- | --- | --- |
| Trial Characteristic | Pearson’s Correlation Coefficient | P value | *n* arms for which data was available for analysis |
| Percentage with ECOG 0 | 0.63 | 0.4 | 11 |
| Percentage with a BRAFV600 mutation (all interventions) | 0.57 | 0.3 | 13 |
| Percentage with LDH ≤ ULN | 0.47 | 0.2 | 14 |
| Age (years) | 0.23 | 0.1 | 13 |
| Percentage with no distant metastases (M0) | 0.35 | 0.1 | 10 |
| Percentage with distant skin, subcutaneous or nodal metastases (M1a) | 0.49 | 0.2 | 9 |
| Percentage with lung metastases (M1b) | 0.49 | 0.2 | 9 |
| Percentage with all other visceral metastases (M1c) | -0.42 | 0.2 | 14 |
| Percentage of patients with grade 3+ adverse events | 0.14 | 0.0 | 9 |
